# Editorial: Mitochondria in Renal Health and Disease, Volume II

**DOI:** 10.3389/fphys.2021.818421

**Published:** 2022-01-10

**Authors:** Egor Plotnikov, Giuliano Ciarimboli

**Affiliations:** ^1^Belozersky Institute of Physico-Chemical Biology, Lomonosov Moscow State University, Moscow, Russia; ^2^Experimental Nephrology, Medicine Clinic D, Münster University Hospital, Münster, Germany

**Keywords:** acute kidney injury, chronic kidney disease, autophagy, mitophagy, nephrotoxicity, caloric restriction, high-fat diet

Mitochondria are intracellular organelles that participate in a wide range of functions besides producing energy. Particularly, mitochondria are also the main storage place for several ions, including metals. However, excessive accumulation of certain metals in the renal mitochondria leads to their dysfunction and the development of acute kidney injury (AKI). In the review by Thévenod et al. the authors summarized the findings showing the role of mitochondrial transporters for iron and cadmium transport and accumulation in kidney mitochondria, leading to nephrotoxicity and AKI.

Mitochondria have developed a variety of quality control systems to cope with internal and external stresses. These systems include control of mitochondrial biogenesis, dynamics, protein quality, and removal. Of special importance is mitochondrial removal, which is accomplished by mitophagy. Mitophagy selectively sequesters damaged or depolarized mitochondria into autophagosomes for subsequent lysosomal degradation.

Impaired mitophagy contributes to the pathogenesis of several human diseases, including diabetic kidney disease (DKD). Loss of mitochondrial quality control disrupts the homeostasis of mitochondria, thus damaging the kidney. A review by Dai et al. summarized advances in understanding the role of mitochondrial biogenesis, dynamics, mitophagy, and protein quality control for the function of kidney mitochondria and their impact on molecular mechanisms related to DKD.

Loss of mitochondrial quality control is also observed during ischemia-reperfusion (IR) injury of the kidneys. Defective removal of damaged mitochondria associated with renal IR promotes kidney injury. The negative effects of a high-fat diet, such as increased oxidative stress and mitochondrial dysfunction caused by IR, are discussed in the article by Prem and Kurian. The authors showed that consumption of a high-fat diet caused significant changes in rat blood chemistry, compromised renal function, and deteriorated mitochondria.

While high-fat diet consumption alters the normal renal function, dietary restriction (DR) was shown to ameliorate AKI and chronic kidney disease. The protective properties of DR are mediated by a range of signaling pathways associated with adaptation to reduced nutrient intake. The adaptation is accompanied by autophagy activation, improvement of mitochondria function, and other metabolic changes. However, with age, the gain of DR-mediated protective effects decreases. In the review by Andrianova et al. authors discuss possible reasons for the decline in nephroprotective mechanisms of DR during aging.

Age-related or hereditary dysfunction of the autophagic-lysosomal system leads to the accumulation of misfolded protein aggregates and dysfunctional mitochondria. Pathological protein aggregates can also lead to profound cellular damage in the kidney. The review by Forst et al. focuses on two autosomal dominant forms of renal Fanconi's syndrome. One of them is associated with the accumulation of unique aggregates of a mutant glycine amidinotransferase (GATM) protein form directly in mitochondria. The half-life of the mutant GATM protein present in fibrils was considerably increased, suggesting impaired degradation of fibrillary protein and whole mitochondria by mitophagy.

Thus, mitochondrial quality control is a promising therapeutic target. In this regard, sirtuin-3 (SIRT3) is a mitochondrial NAD^+^ dependent deacetylase that regulates mitochondrial quality. Suliman et al. demonstrated that ANXA1sp, a tripeptide mimetic of the annexin A1, upregulates SIRT3 and improves markers of mitophagy and limits those of mitochondrial fission.

However, as no effective clinically applied treatments aiming at improving mitochondrial quality are available yet, a better understanding of its regulatory mechanisms is necessary. Yang et al. demonstrated that ING2, a member of the inhibitor of growth (ING) family which is well-known as a tumor suppressor, controls mitochondrial respiration via modulating mitochondrial ribosomal protein L12 (MRPL12) ubiquitination in renal tubular epithelial cells (TECs). ING2 induced elevation of MRPL12 protein was not accompanied by an increase of MRPL12 mRNA suggesting that a mitochondrial MRPL12 protein quality control and removal mechanism might be involved. The authors' study showed that ING2 may be a potential target for interventions in mitochondrial injury-associated pathologies.

## Conclusion

Maintaining mitochondria functionality is the key to maintaining healthy state of tissues and of the whole organism. The pivotal mechanism involved in maintaining mitochondria function is the quality control system through the processes of autophagy and mitophagy. The articles collected in the Research Topic show several ways to affect quality control, either worsening or improving it. Understanding mechanisms of kidney quality control could provide a targeted approach to maintain renal function under damaging conditions.

## Author Contributions

All authors listed have made a substantial, direct, and intellectual contribution to the work and approved it for publication.

## Funding

The work was partially supported by Russian Science Foundation (grant #21-75-30009 to EP) and the Deutsche Forschungsgemeinschaft (CI107/11-1 to GC).

## Conflict of Interest

The authors declare that the research was conducted in the absence of any commercial or financial relationships that could be construed as a potential conflict of interest.

## Publisher's Note

All claims expressed in this article are solely those of the authors and do not necessarily represent those of their affiliated organizations, or those of the publisher, the editors and the reviewers. Any product that may be evaluated in this article, or claim that may be made by its manufacturer, is not guaranteed or endorsed by the publisher.

